# Nfa34810 Facilitates *Nocardia farcinica* Invasion of Host Cells and Stimulates Tumor Necrosis Factor Alpha Secretion through Activation of the NF-κB and Mitogen-Activated Protein Kinase Pathways via Toll-Like Receptor 4

**DOI:** 10.1128/IAI.00831-19

**Published:** 2020-03-23

**Authors:** Xingzhao Ji, Xiujuan Zhang, Heqiao Li, Lina Sun, Xuexin Hou, Han Song, Lichao Han, Shuai Xu, Xiaotong Qiu, Xuebing Wang, Ningwei Zheng, Zhenjun Li

**Affiliations:** a State Key Laboratory for Infectious Disease Prevention and Control, National Institute for Communicable Disease Control and Prevention, Chinese Center for Disease Control and Prevention, Beijing, China; b Department of Endocrinology, Beijing Chaoyang Hospital, Capital Medical University, Beijing, China; c School of Laboratory Medicine and Life Sciences, Wenzhou Medical University, Wenzhou, Zhejiang, China; d Department of Medicine, Tibet University, Lhasa, Tibet, China; e CAS Key Laboratory of Pathogenic Microbiology and Immunology, Institute of Microbiology, Chinese Academy of Sciences, Beijing, China; Stanford University

**Keywords:** *Nocardia farcinica*, Nfa34810, invasion, MAPK, NF-κB, TLR4, tumor necrosis factor

## Abstract

The mechanism underlying the pathogenesis of *Nocardia* is not fully known. The Nfa34810 protein of Nocardia farcinica has been predicted to be a virulence factor. However, relatively little is known regarding the interaction of Nfa34810 with host cells, specifically invasion and innate immune activation. In this study, we aimed to determine the role of recombinant Nfa34810 during infection. We demonstrated that Nfa34810 is an immunodominant protein located in the cell wall. Nfa34810 protein was able to facilitate the uptake and internalization of latex beads coated with Nfa34810 protein into HeLa cells. Furthermore, the deletion of the *nfa34810* gene in *N. farcinica* attenuated the ability of the bacteria to infect both HeLa and A549 cells. Moreover, stimulation with Nfa34810 triggered macrophages to produce tumor necrosis factor alpha (TNF-α), and it also activated mitogen-activated protein kinase (MAPK) and nuclear factor κB (NF-κB) signaling pathways by inducing the phosphorylation of ERK1/2, p38, JNK, p65, and AKT in macrophages. Specific inhibitors of ERK1/2, JNK, and NF-κB significantly reduced the expression of TNF-α, which demonstrated that Nfa34810-mediated TNF-α production was dependent upon the activation of these kinases. We further found that neutralizing antibodies against Toll-like receptor 4 (TLR4) significantly inhibited TNF-α secretion. Taken together, our results indicated that Nfa34810 is a virulence factor of *N. farcinica* and plays an important role during infection. Nfa34810-induced production of TNF-α in macrophages also involves ERK, JNK, and NF-κB via the TLR4 pathway.

## Abstract (precis)

The mechanism underlying the pathogenesis of *Nocardia* is not fully known. The Nfa34810 protein of Nocardia farcinica has been predicted to be a virulence factor. However, relatively little is known regarding the interaction of Nfa34810 with host cells, specifically invasion and innate immune activation. In this study, we aimed to determine the role of recombinant Nfa34810 during infection. We demonstrated that Nfa34810 is an immunodominant protein located in the cell wall.

## INTRODUCTION

*Nocardia* spp. are Gram-positive, partially acid-fast, aerobic, catalase-positive intracellular bacteria found in both the soil and fresh water (1). Nocardiosis is typically an opportunistic disorder that causes severe, life-threatening disseminated infections in immunocompromised hosts (2). *Nocardia* infection predominately causes lung, brain, or skin abscesses; however, it can also cause infection in almost all organs by disseminating through the blood, occasionally resulting in fatal outcomes. There are more than 80 *Nocardia* species that have been described in the literature, with 33 species associated with human diseases (3). As the number of immunodeficient patients and use of immunosuppressive drugs have increased, there has been an increase in the number of reported cases of *Nocardia* infection (2).

There are several published studies regarding the mechanism employed by *Nocardia* to cause disease. Beaman et al. found that *Nocardia* could survive and colonize in macrophages by inhibiting the fusion of the phagosome and lysosome, effectively blocking phagosomal acidification and preventing oxidative killing (4, 5). LeWitt et al. showed that culture filtrates of N. asteroides, especially the low-molecular-mass filtrates, were able to cause dopamine depletion and cytotoxicity in PC12 cells (6, 7). Additionally, *N. asteroides* has been shown to inhibit proteasome activity and induce apoptosis in cells. N. cyriacigeorgica was demonstrated to induce apoptotic changes in bovine mammary epithelial cells through a mitochondrial caspase-dependent apoptotic pathway (4, 8). Xia et al. showed that phospholipase C from N. seriolae induced apoptosis in cells (9). Both adhesion and invasion of host cells by intracellular bacteria are important virulence factors in establishing infection. Several studies have indicated that *Nocardia* organisms can adhere to and invade various types of cells, inducing both cellular and tissue damage (10, 11). Bacterial mammalian cell entry (Mce) proteins are encoded by *mce* genes, and *mce1E* is considered a virulence factor of N. farcinica. We have previously shown that *mce1E* facilitates *N. farcinica* invasion of mammalian cells and may be expressed by *N. farcinica* during infection (12).

Macrophages are the first line of defense against pathogens and play an important role in innate immunity. The mitogen-activated protein kinase (MAPK) and nuclear factor κB (NF-κB) signaling pathways are involved in cellular regulation and play a critical role in innate immunity by mediating the induction of proinflammatory cytokines, such as tumor necrosis factor alpha (TNF-α), interleukin-6 (IL-6), and IL-1β (13). Our unpublished results show that *Nocardia* can activate both the MAPK and NF-κB signaling pathways, resulting in the phosphorylation and activation of p38 kinase, extracellular-regulated kinase (ERK) 1/2, c-Jun-N-terminal (JNK), p65, and AKT and subsequent production of proinflammatory cytokines (unpublished data). Cholesterol oxidase (ChoD) from *N. erythropolis* was shown to be able to activate p38 mitogen-activated kinase and stimulate the production of IL-10 via Toll-like receptor 2 (TLR2) (14). The activation of TLRs by pathogen-associated molecular patterns (PAMPs) can lead to the activation of MAPK and NF-κB signaling pathways, which is crucial for the modulation of innate immunity (15). Millan-Chiu et al. have shown that TLR2 expression increased in N. brasiliensis-infected tissue, whereas TLR4 expression decreased in the advanced stages of the infection (16), indicating that TLRs play an important role in the immune response against *Nocardia* spp.

There is limited research regarding the virulence factors of *N. farcinica*, and as such, it is critical to understand the molecular details underlying *Nocardia*-host cell interactions. The genomic information of *N. farcinica* indicated that *nfa34810* plays a role in adherence to and invasion of host cells (17). In this study, we assessed the role *nfa34810* plays in facilitating invasion of mammalian host cells by cloning and expressing recombinant Nfa34810 protein and by constructing an *nfa34810* deletion mutant (*Δnfa34810*) in *N. farcinica*. We then analyzed the interactions between Nfa34810 and macrophages and investigated the mechanism by which Nfa34810 regulates the innate immune system. Our results demonstrated that Nfa34810 is located in the cell wall and facilitated *N. farcinica* invasion of mammalian cells. Furthermore, we also showed that Nfa34810 was expressed during *N. farcinica* infection and elicited an antibody response, which shows that this protein has the potential to be used in serological diagnosis for its specificity. Moreover, we demonstrated that Nfa34810 promoted the production of TNF-α in macrophages, which depended on the activation of ERK, JNK, and NF-κB signaling pathways via TLR4. Our results provide insights into the underlying mechanism of *N. farcinica* infection and may aid in the development of therapeutics for *Nocardia* infection.

## RESULTS

### Expression and purification of recombinant Nfa34810 protein.

**FIG 1:**
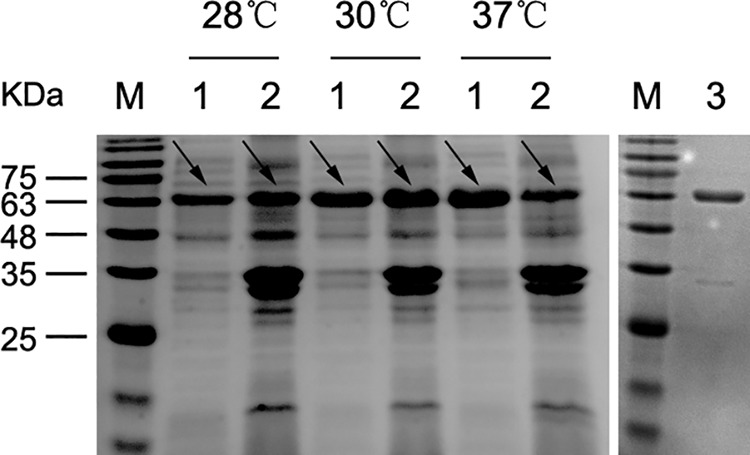
SDS-PAGE analysis of Nfa34810 protein. Lane M, protein markers. Lane 1, supernatant of recombinant E. coli expressing Nfa34810 protein cultured for 4 h with 0.2 mM IPTG. Lane 2, pellet of recombinant E. coli expressing Nfa34810 protein cultured for 4 h with 0.2 mM IPTG. Lane 3, purified Nfa34810 protein.

DNA sequencing confirmed that the pET30a-*nfa34810* recombinant expression vector was constructed successfully. Protein expression induced with 0.2 mM isopropyl-β-d-thiogalactopyranoside (IPTG) was analyzed by SDS-PAGE. The Nfa34810 protein was expressed mainly in the supernatant, and the expression increased with the increase of induction temperature (Fig. 1). Purified Nfa34810 protein presented as a single band, and the purity is about 96.3%.

### Antigenicity of Nfa34810 protein.

**FIG 2:**
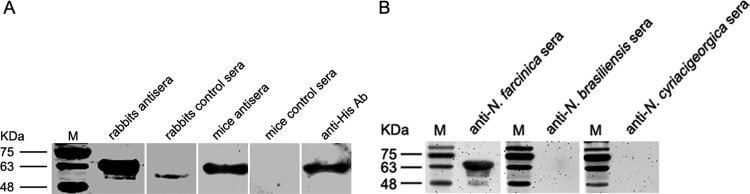
Western blot analysis of the reactivity of Nfa34810 protein with anti-His or antiserum antibodies. (A) Nfa34810 proteins can be recognized by antisera from mice or rabbits infected with *N. farcinica* or anti-His antibodies. (B) The specificity of the antisera for Nfa34810 proteins was analyzed by Western blotting. Nfa34810 protein only reacted with anti-*N. farcinica* sera and not anti-N. brasiliensis or anti-*N. cyriacigeorgica* sera.

We have identified a number of immunogenetic proteins in *N. farcinica* that are not only expressed during infection but also elicit antibody responses during infection (12, 18). Sera from humans infected with *N. farcinica* are not available in our laboratory. However, to account for this, we prepared antisera from different species of animals, including rats and rabbits. As shown in Fig. 2A, recombinant His-Nfa34810 protein was identified by using an anti-His antibody. Recombinant His-Nfa34810 also reacted with antibodies from mice or rabbits infected with *N. farcinica* but not control sera. These results indicated that Nfa34810 is immunogenetic and expressed during infection. To assess the specificity of Nfa34810 with antiserum, we used the antisera from animals infected with N. brasiliensis and *N. cyriacigeorgica*, which have a gene with high similarity to the *nfa34810* gene of *N. farcinica*. As shown in Fig. 2B, Nfa34810 was only recognized by anti-*N. farcinica* sera and was not recognized by anti-N. brasiliensis or anti-*N. cyriacigeorgica* sera.

### Subcellular localization of Nfa34810 proteins.

**FIG 3:**
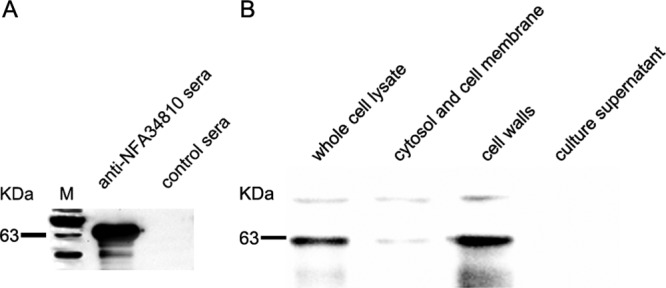
Nfa34810 is localized in the cell wall. (A) The reactivity of Nfa34810 protein with anti-Nfa34810 sera was analyzed using Western blotting. (B) Subcellular localization of Nfa34810 protein was analyzed by Western blotting using anti-Nfa34810 sera at a dilution of 1:4,000.

Genomic analysis predicted that Nfa34810 was a secreted protein (17). To identify the localization of the Nfa34810 protein in *N. farcinica*, we performed cell fractionation and analyzed the cellular localization of Nfa34810 by Western blotting using anti-Nfa34810 sera, which was generated in our laboratory. Nfa34810 protein was identified using antisera from rabbits infected with Nfa34810 (Fig. 3A). As shown in Fig. 3B, the Nfa34810 protein was detected mainly in the cell wall fraction and whole-cell lysate, indicating that Nfa34810 is associated with the cell wall and is not a secreted protein.

### Invasion of HeLa cells by Nfa34810-coated latex beads.

**FIG 4:**
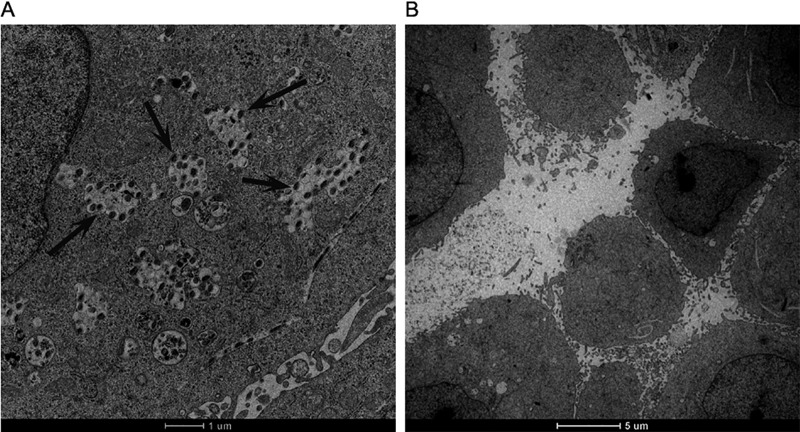
Transmission electron micrographs showing that latex beads coated with Nfa34810 protein were internalized by HeLa cells. (A) HeLa cells were incubated with latex beads coated with Nfa34810 for 24 h, and the latex beads were internalized completely by the HeLa cells (arrows). Bar, 1 μm. (B) Latex beads without protein coating were not visible in cells. Bar, 5 μm.

Transmission electron microscopy (TEM) has been used in several studies to demonstrate that protein-coated latex beads are internalized by HeLa cells (12, 19). As shown in Fig. 4, internalized latex beads coated with Nfa34810 were observed within vacuolar compartments after 24 h of incubation, whereas uncoated latex beads were not internalized (Fig. 4).

### Nfa34810 facilitated *N. farcinica* invasion of HeLa and A549 cells.

**FIG 5:**
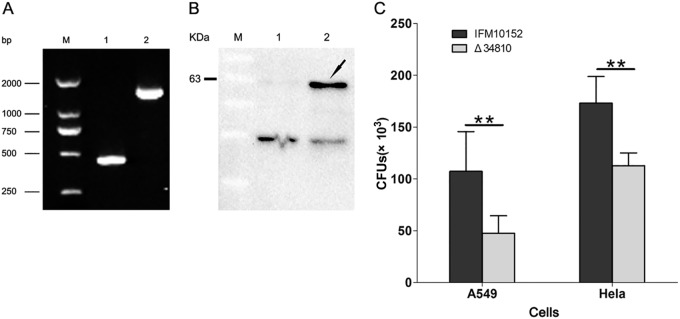
Ability of wild-type and *Δnfa34810* mutant *N. farcinica* to invade HeLa or A549 cells was assessed by invasion assays. (A) Confirmation of *Δnfa34810* mutant by PCR. Lane M, markers. Lane 1, gene amplification fragments from *Δnfa34810* mutant. Lane 2, gene amplification fragments from the wild type. (B) Confirmation of *Δnfa34810* mutant by Western blotting. Lane M, markers. Lane 1, the whole protein of mutants strains was detected with anti-Nfa34810. Lane 2, the whole protein of wild strains was detected with anti-Nfa34810. (C) Invasion of HeLa or A549 cells by *N. farcinica* wild type and *Δnfa34810* mutant. HeLa or A549 cells were infected with *N. farcinica* wild type or *Δnfa34810* mutant for 1 h at an MOI of 10, and then the concentration of intracellular bacteria was determined. The assays were performed in triplicate, and the results are expressed as the means ± SD. **, *P* < 0.01.

In the above-described assays, we demonstrated that Nfa34810 can facilitate latex bead invasion of HeLa cells. In this study, the *Δnfa34810* mutant with an in-frame deletion was constructed to investigate the role of Nfa34810 during host cell invasion. The deletion of *nfa34810* in *N. farcinica* was confirmed by PCR and DNA sequencing. The *nfa34810* gene was deleted completely in *N. farcinica* (Fig. 5A and B), and the mutant strain demonstrated the same growth rate as the wild-type strain (data not shown). Wild-type and *Δnfa34810* strains were used in invasion assays with HeLa and A549 cells. The results showed that invasion of the *nfa34810* mutant into HeLa cells or A549 cells was significantly reduced compared to that of *N. farcinica* wild-type bacteria (*P* < 0.01) (Fig. 5C). These results demonstrated that Nfa34810 is a necessary component for the efficient entry of *N. farcinica* into host cells.

### Nfa34810 stimulated the production of TNF-α by RAW264.7 macrophages.

**FIG 6:**
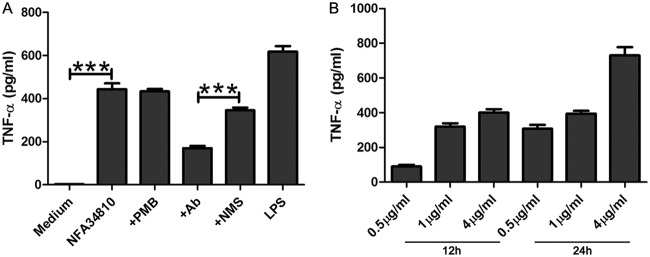
Nfa34810 induced TNF-α secretion from RAW264.7 cells. (A) RAW264.7 cells were cultured in the presence of Nfa34810 (1 μg/ml) for 18 h at 37°C. To exclude the possibility that LPS contaminated the recombinant protein preparation, Nfa34810 was pretreated with an inhibitor of LPS, polymyxin B (+PMB). LPS (100 ng/ml) was used as a positive control. To confirm that the activation of RAW264.7 cells was a result of Nfa34810, the proteins (1 mg/ml) were pretreated with anti-Nfa34810 sera (+Ab, 1:200) or control sera (+NMS, 1:200). Cell culture supernatants were collected, and TNF-α was measured at 18 h. (B) RAW264.7 cells were cultured in the presence of various concentrations of Nfa34810 (0.5, 1, or 4 μg/ml). Cell culture supernatants were collected at 12 or 24 h, and the levels of TNF-α were measured by ELISA. Data are expressed as the means ± SD from three separate experiments. ***, *P* < 0.001.

To determine the effect of Nfa34810 on the activation of the innate immune system, RAW264.7 macrophages were treated with various concentrations of Nfa34810 for 18 h. The culture supernatants then were collected, and the levels of TNF-α, IL-6, IL-1β, and IL-10 in the culture supernatants were measured by enzyme-linked immunosorbent assay (ELISA). RAW264.7 cells exposed to Nfa34810 significantly upregulated the expression of TNF-α in a dose-dependent manner (Fig. 6B). However, Nfa34810 failed to stimulate the upregulation of IL-6, IL-1β, and IL-10 expression at the protein level in macrophages (data not shown). To exclude the possibility that lipopolysaccharide (LPS) contamination contributed to the induction of TNF-α production in these cells, Nfa34810 was pretreated with polymyxin B. Our results demonstrated that polymyxin B did not alter the Nfa34810-induced TNF-α cytokine production (Fig. 6A). Furthermore, TNF-α production was significantly reduced in macrophages stimulated with Nfa34810 and blocked with anti-Nfa34810 antibodies (Fig. 6A), indicating that the activation of the macrophages was attributed to Nfa34810.

### Nfa34810 activated the MAPK and NF-κB signaling pathways.

**FIG 7:**
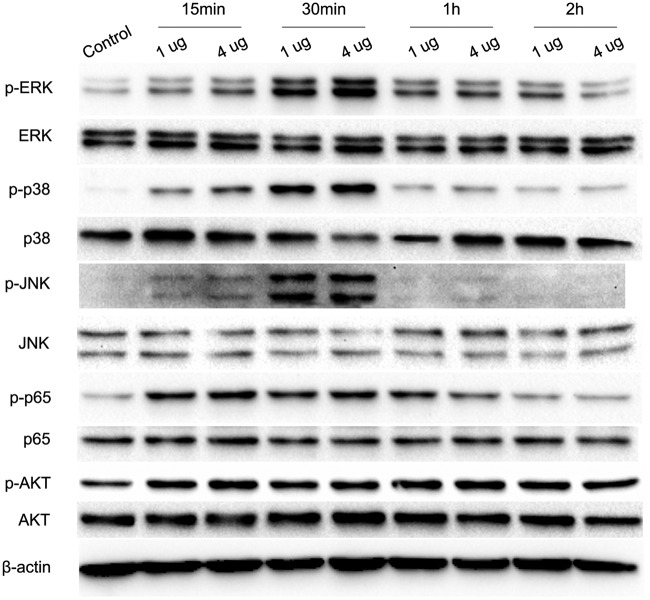
Nfa34810 induced the phosphorylation of ERK, p38, JNK, P65, and AKT. RAW264.7 cells were incubated with Nfa34810 (1 and 4 μg/ml) for the indicated times (15 to 120 min), cell lysates were prepared, and the phosphorylation/activation status of ERK1/2, p38, JNK, p65, and AKT was analyzed by Western blotting. Data are representative of at least three independent experiments.

Many cellular responses to stimulation are dependent on MAPK and NF-κB signaling pathways. To investigate whether MAPK and NF-κB were involved in the innate immune responses activated by Nfa34810, we examined the effect of Nfa34810 on MAPK or NF-κB activation using Western blotting. RAW264.7 cells were stimulated with Nfa34810 (1 and 4 μg/ml), and the phosphorylation status of p38, JNK, ERK1/2, AKT, and p65 was detected after stimulation with Nfa34810. Our results showed that Nfa34810 stimulation resulted in phosphorylation of p38, JNK, ERK1/2, p65, and AKT after 15 to 30 min (Fig. 7). The peak phosphorylation of p38, JNK, and ERK1/2 occurred within 30 min and returned to baseline after 1 h of stimulation with Nfa34810. Nfa34810 also induced phosphorylation of p65 and AKT after 15 min of stimulation, and the phosphorylation of AKT lasted for at least 2 h after stimulation.

### Nfa34810-induced TNF-α expression depended on the activation of ERK1/2, JNK, and NF-κB signaling.

**FIG 8:**
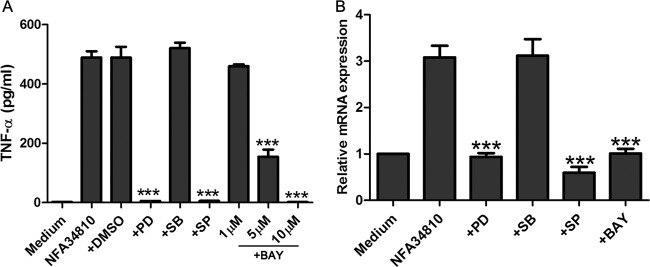
Nfa34810-mediated cytokine expression was dependent on ERK1/2, JNK, and NF-κB. (A) RAW264.7 cells were pretreated for 1 h with PD98059 (+PD; 20 μM), SB203580 (+SB; 10 mM), SP600125 (+SP; 20 μM), BAY 11-7082 (+BAY; 1, 5, and 10 μM), or dimethyl sulfoxide (0.01%) and then stimulated with Nfa34810 (1 mg/ml). After incubation, supernatants were harvested, and levels of TNF-α were measured by ELISA. (B) RAW264.7 cells were pretreated for 1 h with PD98059 (+PD; 20 μM), SB203580 (+SB; 10 mM), SP600125 (+SP; 20 μM), or BAY 11-7082 (+BAY; 5 μM) and then stimulated with Nfa34810 (1 mg/ml) for 6 h. RNA was extracted, and the relative mRNA level of TNF-α in the presence of each inhibitor was calculated and expressed as fold change relative to the control. Data are expressed as the means ± SD from three separate experiments. ***, *P* < 0.001.

To determine the role of JNK, p38, ERK1/2, and NF-κB in Nfa34810-induced TNF-α production, RAW264.7 cells were pretreated with 20 μM PD98059 (MEK inhibitor), 20 μM SP600125 (JNK inhibitor), 10 μM SB203580 (p38 MAPK inhibitor), or 1, 5, or 10 μM BAY 11-7082 (NF-κB inhibitor) for 1 h. Nfa34810 (1 μg/ml) then was added to the culture, and TNF-α production was measured by ELISA after 18 h. As shown in Fig. 8A, TNF-α production was significantly inhibited by the ERK1/2 and JNK inhibitors but was not affected by the p38 inhibitor. Additionally, TNF-α production was inhibited by the NF-κB inhibitor in a dose-dependent manner. Similar results were obtained when the relative mRNA expression of TNF-α was assessed (Fig. 8B). These results indicated that the ERK1/2, JNK, and NF-κB signaling pathways played a critical role in Nfa34810-induced TNF-α production.

### Nfa34810 stimulated the production of TNF-α by macrophages via a TLR4-dependent mechanism.

**FIG 9:**
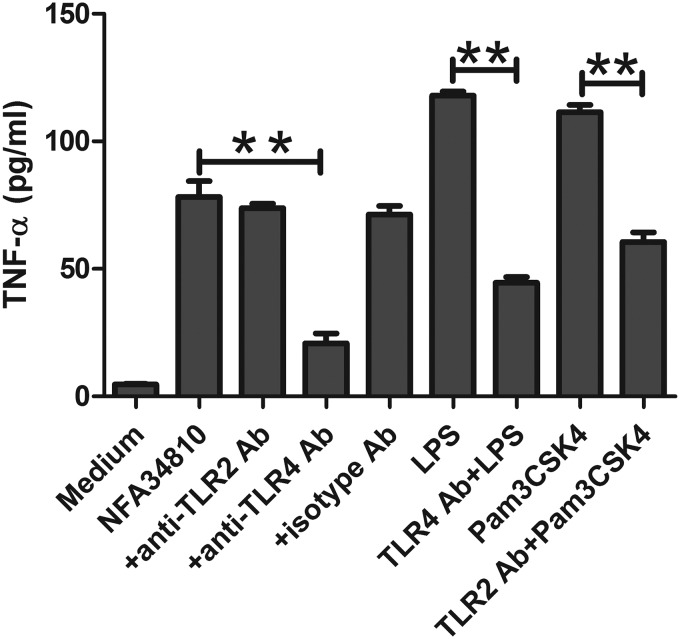
TLR4 is required for the secretion of TNF-α by Nfa34810-induced macrophages. THP-1 cells were pretreated for 1 h with anti-TLR2 (10 μg/ml), anti-TLR4 (10 μg/ml), or isotype control antibodies (10 μg/ml) before stimulation with Nfa34810, LPS, or Pam3CSK4. The levels of TNF-α in the cell culture supernatants were measured by ELISA after 18 h of stimulation. Data are expressed as the means ± SD from three independent experiments. **, *P* < 0.001.

To identify which TLR was involved in the Nfa34810-induced immune activation, THP-1 cells were pretreated with or without blocking antibody against human TLR2, TLR4, or an isotype control antibody for 1 h prior to stimulation with Nfa34810, LPS, or Pam3CSK4. The production of TNF-α was measured 18 h after stimulation. LPS and Pam3CSK4 were used as positive controls to demonstrate the blocking effects of anti-TLR4 and anti-TLR2 antibodies. As shown in Fig. 9, blockade of TLR4 signals significantly decreased the production of TNF-α after Nfa34810 stimulation (*P* < 0.001; ∼70% inhibition at 10 μg/ml). However, neither the anti-TLR2 nor the isotype antibody affected the cytokine secretion in THP-1 cells. These results indicated that TNF-α production induced by Nfa34810 protein is mediated by TLR4 signals.

## DISCUSSION

The virulence factors of the intracellular pathogenic bacterium *N. farcinica* are not fully understood. The initial step in the pathogenesis of intracellular pathogens is invasion of host cells. We have found that Mce1E, derived from *N. farcinica*, plays an important role in the process of host cell invasion (12). In this study, our results demonstrated that Nfa34810 of *N. farcinica* is a virulence factor and is also involved in the infection process.

The Nfa34810 protein is recognized by anti-*N. farcinica* sera, which indicates that this protein is an immunodominant protein. The sera from patients infected with *N. farcinica* are not available in our laboratory, and as such, we used antisera from mice or rabbits to assess the antigenicity of Nfa34810 in this study. We found that Nfa34810 reacted with sera from mice or rabbits infected with *N. farcinica*, which indicated that this protein was able to elicit an antibody response during infection. We compared the *nfa34810* gene sequence to sequence databases using NCBI and found that the gene has good specificity for *N. farcinica*. Anti-N. brasiliensis and -N. cyriacigeorgica sera were used to verify that this protein was specific to *N. farcinica*. Our results showed that Nfa34810 was only recognized by anti-*N. farcinica* sera and not anti-N. brasiliensis or anti-*N. cyriacigeorgica* sera, indicating that this protein has clinical applications in the serological diagnosis of *N. farcinica* infection. Future studies are needed to analyze the B cell epitope of this protein to use it as a screening tool in the clinical diagnosis of *N. farcinica* infection.

Genomic analysis predicted that Nfa34810 may be involved in cell invasion (17). To determine if Nfa34810 was indeed involved in host cell invasion, the subcellular localization of Nfa34810 needed to be confirmed. Our results showed that Nfa34810 was associated with the cell wall and likely available for interaction with host components. In our previous study, latex beads coated with Mce1E were internalized by HeLa cells (12). El-Shazly et al. showed that Mce3A and Mce3E derived from Mycobacterium tuberculosis facilitated the uptake and internalization of latex beads by HeLa cells (19). In this study, latex beads coated with purified Nfa34810 protein were internalized by HeLa cells, indicating that the Nfa34810 protein plays a role in invasion. To further confirm this, the *Δnfa34810* mutant was constructed. As shown in Fig. 5C, the deletion of *nfa34810* attenuated the invasion of *N. farcinica* into HeLa or A549 cells. Bacteria can exploit cell surface receptors for entry into mammalian cells through a receptor-dependent internalization mechanism. Liu et al. showed that Mce3C of Mycobacterium tuberculosis exploits the integrin-mediated signaling cascade for mammalian cell entry (20). It will be interesting to identify the interacting mammalian proteins that might serve as receptors for the Nfa34810 protein, as this could provide a better understanding of the invasion mechanisms of *N. farcinica*.

In the present study, we found that Nfa34810 protein was associated with the cell wall and elicited the production of specific antibodies. Thus, Nfa34810 plays an important role in the interaction with nonphagocytic cells. To fully understand its function, we further analyzed the role of this protein in its interaction with macrophages. Macrophages play a critical role in both innate and adaptive immunity, which is required to control intracellular bacterial infections (21). The innate immune system is important, since *Nocardia* species evasion is mediated through uptake and intracellular survival in macrophages of the host (22). Coulombe et al. have found that *N. asteroides* could activate innate immunity via the host sensor NOD2 (23). Salinas-Carmona et al. reported that N. brasiliensis modulates macrophage cytokine production, which serves as a pathogenic mechanism during infection (24). The proinflammatory cytokines produced by macrophages responding to *N. farcinica* include TNF-α, IL-6, IL-1β, and IL-12 p40. Bacterial proteins are known to stimulate the production of proinflammatory cytokines from macrophages, but to the best of our knowledge, this is the first time that a protein of *Nocardia* spp. was shown to be involved in regulation of the innate immune system.

Our results showed that recombinant Nfa34810 protein derived from *N. farcinica* was able to stimulate RAW264.7 cells to significantly increase the expression of TNF-α at both the protein and mRNA levels in a dose-dependent manner. The production of TNF-α was attenuated or blocked by anti-Nfa34810 sera, which supported that the effect was elicited specifically by Nfa34810. Interestingly, we found that the recombinant Nfa34810 protein also induced the mRNA expression of IL-6 and IL-12 p40 by RAW264.7 cells (data not shown), but the protein of these cytokines was not detected by ELISA. Both the MAPK and NF-κB signaling pathways are central to the expression of cytokines, such as TNF-α, IL-6, IL-1β, and IL-12 p40, in the inflammatory response (13). ERK, JNK, and p38 can be independently or simultaneously activated to induce the expression of proinflammatory cytokines (25, 26). The phosphorylation of p65, which belongs to the NF-κB signaling cascade, is important for immune responses, as it also induces the expression of proinflammatory cytokines (27). Our results show that Nfa34810 protein rapidly induced the phosphorylation of p38, JNK, ERK1/2, p65, and AKT. To further dissect the role of phosphorylated MAPK and NF-κB in triggering the production of TNF-α, highly specific inhibitors of p38 (SB203580), ERK1/2 (PD98059), JNK (SP600125), and NF-κB (BAY-117082) were used to suppress the activation of these kinase cascades. Our results showed that ERK1/2 and JNK inhibitors reduced the production of TNF-α at the protein and mRNA levels, and expression of TNF-α was significantly suppressed by the NF-κB inhibitor BAY 11-7082 in a dose-dependent manner. Interestingly, p38 was activated by Nfa34810, but the p38 inhibitor did not influence the expression of TNF-α. We found that the mRNA levels of IL-6 and IL-12 p40 were upregulated after stimulation with Nfa34810 protein, and phosphorylation of p38 may be connected to the upregulated expression of these two proinflammatory cytokines. These data highlight the important role of the JNK, ERK1/2, and NF-κB pathways in the Nfa34810-induced production of TNF-α by macrophages and further indicate that the effects of Nfa34810 protein on cytokine production are mediated by both the MAPK and NF-κB signaling pathways. Our results further demonstrate that Nfa34810, not LPS contamination, induced the expression of proinflammatory cytokines and activation of signaling pathways.

TLRs, especially TLR2 and TLR4, play a critical role in the activation of MAPK pathways and phosphorylation of NF-κB in macrophages. The expression of proinflammatory cytokines, such as TNF-α, IL-1β, IL-6, and IL-12, is mediated via TLR2 or TLR4 (28, 29). Some bacterial components, such as bacterial proteins or lipoproteins, can promote the expression of proinflammatory cytokines by modulating TLR2 or TLR4 signaling (30–32). A previous study showed that TLR2 and TLR4 were expressed in tissue infected with *Nocardia* during the early stages of infection (16). To investigate if the upregulation of TNF-α expression induced by Nfa34810 protein signaled through TLR2 or TLR4 receptors, human anti-TLR2/4 antibodies were used to block the receptors in cell lines. Our results showed that the Nfa34810-induced production of TNF-α was significantly inhibited by the blocking effect of the anti-TLR4 antibody, which suggested that TLR4 was essential for the production of TNF-α by macrophages after stimulation with Nfa34810 protein.

In summary, our results demonstrated that Nfa34810, an immunodominant protein and virulence factor of *N. farcinica*, was expressed during infection and able to elicit an antibody response from the host cells, which may benefit the diagnostic strategies for *N. farcinica* infection. Further, Nfa34810 facilitated *N. farcinica* invasion of host mammalian cells and regulated the innate immune response as a potent TLR4 agonist. Nfa34810 also activated the MAPK and NF-κB signaling pathways and induced expression of the inflammatory cytokine TNF-α via TLR4. Thus, our studies provide a better understanding of the molecular mechanisms underlying the interactions between *N. farcinica* and the host and provide insights needed for the prevention and treatment of *N. farcinica* infection.

## MATERIALS AND METHODS

### Bacterial strains, cells, and culture conditions.

The standard laboratory *N. farcinica* strain IFM10152 was purchased from the German Resource Centre for Biological Materials and cultured in brain heart infusion (BHI) medium (Oxoid, China) at 37°C. Escherichia coli DH5α and BL21(DE3) cells (TransGen Biotech, Beijing, China) were grown in LB medium at 37°C. All of the cell lines used in this study, including the human epithelial HeLa cells, murine macrophage RAW264.7 cells, and human monocytic THP-1 cells, were maintained in our laboratory. HeLa cells and RAW264.7 cells were grown in Dulbecco’s modified Eagle’s medium (DMEM; Gibco, Gaithersburg, MD, USA), and THP-1 cells were grown in RPMI 1640 medium (Gibco) supplemented with 10% fetal bovine serum (FBS; Gibco) in a humidified incubator with 5% CO_2_ at 37°C. The THP-1 cells were differentiated into macrophages by incubation with 100 ng/ml phorbol myristate acetate (PMA; Sigma, Germany) for 24 h.

### Plasmids, antibodies, and reagents.

The pET30a vector (constructed in our laboratory) was used to express *N. farcinica* Nfa34810 in E. coli. The suicide plasmid pk18mobsacB (constructed in our laboratory) was used to construct the *nfa34810* deletion mutant. The following antibodies were used in this study: anti-p-Jnk and -JNK (4668; Cell Signaling), anti-p-ERK 1/2 and -ERK 1/2 (4370; Cell Signaling), anti-p-p38 and -p38 (4511; Cell Signaling), anti-p-p65 and -p65 (3033; Cell Signaling), anti-p-AKT and -AKT (4060; Cell Signaling), anti-β-actin (TransGen Biotech), anti-TLR4 (NB100-56727; Novus Biological, USA), anti-TLR2 (NB100-56726; Novus Biological, USA), and IgG_2A_ isotype control (MAB003; R&D Systems, USA). Human and mouse TNF ELISA kits were used in this study (BD, USA). The following reagents were purchased from Sigma-Aldrich (St. Louis, MO, USA): p38 inhibitor (SB203580), ERK1/2 inhibitor (PD98059), JNK inhibitor (SP600125), and NF-κB inhibitor (BAY 11-7082). An endotoxin removal kit was purchased from GenScript (Nanjing, China).

### Cloning, expression, and purification of recombinant Nfa34810.

*N. farcinica nfa34810* was synthesized by Sangon Biotech, cloned in pET30a to generate pET30a-*nfa34810*, and then transformed into *E. coli* DH5α. Subsequently, *E. coli* DH5α was grown on LB agar in the presence of 50 μg/ml kanamycin (TransGen Biotech) to select for positive clones. The recombinant plasmids were sequenced and then transformed into E. coli BL21 cells.

Recombinant E. coli BL21 cells were cultured at 37°C in LB medium containing 50 μg/ml kanamycin and induced with 0.2 mM isopropyl β-d-1-thiogalactopyranoside (IPTG; TransGen Biotech) at 37°C, as previously described (12). The bacteria then were sonicated and centrifuged at 12,000 rpm at 4°C for 20 min. The pellet and culture supernatant were analyzed by SDS-PAGE. The recombinant proteins then were purified using the His·Bind purification kit (Novagen, Germany), according to the manufacturer’s instructions. Endotoxin was removed from the purified recombinant Nfa34810 preparations using a ToxinEraser endotoxin removal kit (GenScript). Protein concentrations were determined by bicinchoninic acid (BCA) assay and stored at −80°C until used.

### Determination of the immunogenicity of the Nfa34810 protein.

To determine if Nfa34810 protein was expressed by *N. farcinica* and elicited the production of antibodies during infection, Western blot analysis was performed using a previously published protocol, with slight modifications (12). The recombinant Nfa34810 protein was transferred onto polyvinylidene fluoride membranes (Merck, Germany) and blocked with 5% skim milk overnight at 4°C. Anti-*N. farcinica* serum from mice or rabbits was used as the primary antibody, and anti-rabbit IgG (Beyotime Biotechnology, Shanghai, China) or anti-mouse IgG (SouthernBiotech, Birmingham, AL, USA) antibody was used as the secondary antibody. The mouse antiserum of N. brasiliensis and *N. cyriacigeorgica*, produced in our laboratory, was used to analyze the cross-reactivity of Nfa34810 protein with antibodies produced by infection with other *Nocardia* species.

### Subcellular localization of Nfa34810 protein.

The subcellular location of Nfa34810 was determined using the method previously described (33, 34). Briefly, *N. farcinica* was cultured in BHI for 16 to 18 h. Culture filtrate protein was precipitated using methanol and trichloromethane, and the cells were lysed by sonication. Unlysed cells were removed by centrifuging the samples at 5,000 × *g* for 45 min at 4°C. The supernatant then was subjected to ultracentrifugation at 27,000 × *g* for 1 h at 4°C to separate the cell membrane and cytosol fraction. The pellet contained the cell wall fraction. Equal protein amounts (8 μg) from each fraction were subjected to Western blotting using anti-Nfa34810 rabbit serum as the primary antibody. Briefly, the antiserum was prepared by subcutaneously immunizing rabbits with Nfa34810 supplemented with an equal volume of Freund’s incomplete adjuvant three times every 2 weeks. Serum was collected 7 days after the last immunization. Antiserum titers were determined by ELISA.

### Invasion assays.

The genomic information of *N. farcinica* IFM10152 suggests that *nfa34810* plays a role in adhesion and invasion (17). To verify whether this gene confers invasive functions to *N. farcinica*, recombinant Nfa34810 protein was used in the corresponding assays. Briefly, 5 μl of latex beads (4%, wt/vol, 0.3-μm diameter; Thermo Fisher Scientific, Waltham, MA, USA) was coated with 60 μg of Nfa34810 protein in 1 ml of phosphate-buffered saline (PBS) and then incubated for 2 h at 37°C. Latex beads that were not coated with protein served as the control. Briefly, 2 × 10^5^ HeLa cells were seeded into a 24-well plate and grown until confluence. Latex beads coated with Nfa34810 protein (200 μl) or latex beads alone (200 μl) were added to the HeLa cell monolayers and incubated at 37°C for 24 h. The monolayers then were washed three times with PBS and processed for electron microscopy. The cells were fixed with 2% glutaraldehyde, postfixed in 1% osmium tetroxide, and dehydrated with an increasing concentration gradient of ethanol solutions. Ultrathin sections of the cell samples were cut and then examined by transmission electron microscopy (TEM; HT7700; Hitachi, Japan).

### Construction of deletion mutants.

The *nfa34810* in-frame deletion (*Δnfa34810*) mutant was constructed by using homologous recombination. A DNA fragment (900 bp with an EcoRI site) upstream of *nfa34810* and another fragment (1,000 bp with a HindIII site) downstream of *nfa34810* were amplified by PCR using primers Dnfa-1F/Dnfa-1R and Dnfa-2F/Dnfa-2R (Table 1). These two fragments were ligated by bypass PCR amplification using primers Dnfa-1F/Dnfa-2R (Table 1) to generate the *nfa34810* deletion fragment, which was subsequently inserted into the vector pK18mobsacB to obtain pK18mobsacB-*Δnfa34810*. Competent *N. farcinica* was prepared by washing cells grown to logarithmic phase three times with ice-cold water. *N. farcinica* then was resuspended in ice-cold 10% glycerol (35). The pK18mobsacB-*Δnfa34810* plasmid was transformed into *N. farcinica* by electroporation using the following standard settings: 2.5 kV, 1,000 Ω, and 25 μF. After pulsing with a Gene Pulser Xcell apparatus (Bio Rad, USA), the electroporated cells were added to 900 μl of BHI broth and incubated for 2 h at 37°C. The transformed cells then were plated onto BHI agar plates containing 100 μg/ml of neomycin and incubated at 37°C for 2 days. The positive colonies were selected and plated onto BHI agar plates containing 20% sucrose to select for the loss of the genome-integrated *sacB*-containing plasmid. The deletion of *nfa34810* was confirmed by PCR and DNA sequencing using the Dnfa-F and Dnfa-R primer pair (Table 1). The deletion of Nfa34810 was confirmed by Western blotting.

**TABLE 1:** Primers used for construction of deletion mutants and RT-PCR

Primer	Sequence (5′ to 3′)
Dnfa-1F	CAGAGAATTCCTGTGCTCTGGCAGTGTTGGAT
Dnfa-1R	TGGAATTCGGCGCTCGCGCACCTCCAAGGACTCGA
Dnfa-2F	AGTCCTTGGAGGTGCGCGAGCGCCGAATTCCACCG
Dnfa-2R	TACCAAGCTTGTCCTGCGGCACCACGTAGTCGC
Dnfa-F	GGGTTCCGGCTTGTCCTTT
Dnfa-R	TTCCAGACGGTCGGTATGTTTT
GAPDH-F	AACGACCCCTTCATTGAC
GAPDH-R	TCCACGACATACTCAGCAC
TNF-F	ATGAGCACAGAAAGCATGATC
TNF-R	TACAGGCTTGTCACTCGAATT

### Invasion of HeLa cells and A549 cells by *N. farcinica*.

*N. farcinica* wild-type and *Δnfa34810* strains were used to further confirm the invasion function of Nfa34810. A549 or HeLa cells were inoculated into DMEM supplemented with 10% fetal bovine serum, seeded at a density of 2 × 10^5^ cells/well in 24-well plates, and grown until a cell monolayer formed. The cells were resuspended in DMEM supplemented with 2% FBS and then were infected with the wild-type or *Δnfa34810* strain of *N. farcinica* for 1 h at a multiplicity of infection (MOI) of 10. After incubation, the cells were washed three times with sterile PBS to remove extracellular bacteria and then were cultured in fresh medium containing 100 μg/ml amikacin (TransGen Biotech) for 2 h. The cells then were washed three times with PBS and then solubilized with 1 ml of sterile water for 20 min at 37°C. The intracellular bacteria were determined by plating serially diluted cultures on BHI plates.

### Cytokine ELISAs.

RAW264.7 cells were seeded into 24-well plates at a density of 2 × 10^5^ cells/well and stimulated with Nfa34810 or LPS (Sigma-Aldrich) for 18 h. To confirm that LPS contamination in the purified aliquot of Nfa34810 did not cause immune cell stimulation, Nfa34810 was preincubated with polymyxin B (a specific inhibitor for LPS; INALCO, USA). To determine if Nfa34810 protein was responsible for the release of proinflammatory cytokines from macrophages, Nfa34810 was preincubated with anti-Nfa34810 serum or control serum for 1 h at 37°C. In experiments designed to block MAPK and NF-κB signaling, RAW264.7 cells were pretreated for 1 h at 37°C with inhibitors of p38 (10 μM), ERK (20 μM), JNK (20 μM), or NF-κB (1, 5, or 10 μM) prior to Nfa34810 exposure as described above. In experiments designed to block TLR signaling, THP-1 cells were pretreated for 1 h at 37°C with an antibody against human TLR2 (10 μg/ml), TLR4 (10 μg/ml), or a IgG isotype-matched control antibody (10 μg/ml). The cell culture supernatants then were collected at 18 h, and the levels of TNF-α were measured using ELISA.

### RT-PCR analysis.

RAW264.7 cells were seeded into 6-well plates at a density of 1 × 10^6^ cells/well and treated for various lengths of time with Nfa34810. Total RNA then was extracted using the RNeasy Plus minikit (Qiagen, Hilden, Germany) according to the manufacturer’s recommended protocol. Potential DNA contamination was removed during the process of RNA extraction. cDNA synthesis was performed at 37°C for 15 min, followed by 85°C for 5 min using the PrimeScript reverse transcription (RT) reagent kit (TaKaRa, Kusatsu, Japan). Real-time PCR was carried out using the SYBR premix *Ex Taq* II reagents (TaKaRa) according to the manufacturer’s instructions. RNA levels of the analyzed genes were normalized to the amount of *GAPDH* present in each sample. The primers (Table 1) for *GAPDH* and *TNF* genes were synthesized by Sangon (Shanghai, China).

### Protein preparation and Western blot analysis.

RAW264.7 cells were seeded into 6-well plates at a density of 1 × 10^6^ cells/well and stimulated with Nfa34810 for various periods of time. The cells then were lysed with radioimmunoprecipitation assay buffer supplemented with phosphatase and protease inhibitors (CWBIO, China) on ice for 30 min. The supernatant was collected after centrifuging at 12,000 × *g* at 4°C for 25 min, and then the protein concentration was measured using a BCA protein assay kit (Tiangen Biotechnology, China). Equal protein concentrations were separated by SDS-PAGE and then transferred to polyvinylidene fluoride membranes (Millipore). After blocking with 5% nonfat dry milk in Tris-buffered saline with Tween 20 (TBST) for 2 h at room temperature, the membranes were incubated overnight at 4°C with primary antibodies, including p-ERK1/2, p-p38, p-JNK, p-p65, p-AKT, NF-κB p-p65, and β-actin. Subsequently, membranes were incubated for 1 h with horseradish peroxidase-conjugated anti-rabbit IgG (Beyotime Biotechnology) or anti-mouse IgG antibodies (SouthernBiotech) and detected using a Western Lightning plus ECL kit (PerkinElmer, USA).

### Statistical analysis.

Results are shown as the means ± standard deviations (SD) from triplicate experiments. Analyses were performed using SPSS 22.0. Group means and standard deviations were compared using Student’s *t* tests. A *P* value of <0.05 was considered statistically significant.
